# Design Strategy for Vulcanization Accelerator of Diphenylguanidine/Cyclodextrin Inclusion Complex for Natural Rubber Latex Foam with Enhancing Performance

**DOI:** 10.34133/2022/9814638

**Published:** 2022-09-01

**Authors:** Wang Zhang, Liwei Lin, Junqiang Guo, Ming Wu, Sumin Park, Hang Yao, Sun Ha Paek, Guowang Diao, Yuanzhe Piao

**Affiliations:** ^1^Department of Applied Bioengineering, Graduate School of Convergence Science and Technology, Seoul National University, Seoul 08826, Republic of Korea; ^2^School of Chemistry and Chemical Engineering, Yangzhou University, Yangzhou, Jiangsu 225002, China; ^3^Department of Neurosurgery, Movement Disorder Center, Seoul National University Hospital, Hypoxia/Ischemia Disease Institute, Cancer Research Institute, Seoul National University College of Medicine, Seoul 03080, Republic of Korea; ^4^Advanced Institutes of Convergence Technology, 145 Gwanggyo-ro, Yeongtong-gu, Suwon-si, Gyeonggi-do, 16229, Republic of Korea

## Abstract

Vulcanization is an essential process to obtain high-performance rubber products. Diphenylguanidine (DPG) is often used as the secondary accelerator in the vulcanization process of natural rubber (NR) latex. However, DPG would make NR latex emulsion exhibit gelation, resulting in the negative vulcanization efficiency. In addition, exposure to DPG might lead to some physiological diseases during the production process of DPG doped NR latex. Hydroxypropyl-*β*-cyclodextrin (HP-*β*-CD) with the hydrophobic interior and hydrophilic exterior has the advantages of good water solubility, high bioavailability, reliable stability, and low toxicity. In this study, the inclusion complex of diphenylguanidine-hydroxypropyl-*β*-cyclodextrin (DPG-HP-*β*-CD) is prepared by ball milling with a host-guest molar ratio of 1 : 1, which has also been applied to the foaming process of NR latex. The mechanical properties of DPG-HP-*β*-CD inclusion complex/natural rubber latex foam (DPG-HP-*β*-CD/NRLF) have been significantly improved, including the tensile strength, elongation at break, hardness, compression set, resilience, and antiaging performance. Further, the usage of DPG has been reduced, leading to the reduction of toxicity and environmental hazards.

## 1. Introduction

Natural rubber (NR) latex products can be divided into four categories: impregnated products, molded products, extrusion products, and foam products. Compared with the first three, natural rubber latex foam (NRLF) is a kind of low-density porous material, with elastic elasticity, water absorption, sound insulation, and shockproof characteristic, which is prepared through physical or chemical foaming process [1–4]. NRLF is widely used in vehicle seats, transportation packaging, furniture, and cosmetic products [5]. Vulcanization is the final process of the preparation of NRLF, which would endow the NRLF with appropriate strength and elasticity [6–8]. The essence of vulcanization process is the formation of crosslinking and chemical bonding between rubber molecules, thus transforming the dispersed rubber molecules into a network structure [9, 10]. Although the vulcanization of rubber has been developed from sulfur vulcanization to organic accelerator vulcanization, but due to the comprehensive consideration of rubber products using sulfur vulcanization and the low sulfur cost, sulfur vulcanization still widely occupies the industrial market [11]. If only sulfur is used for vulcanization, the efficiency would be low, not only the time is long but also the required temperature is high. Hence, people generally use vulcanization accelerators to increase the speed, which promotes the development of the rubber industry. As an important part of vulcanization, vulcanization accelerator, such as diphenylguanidine (DPG) and dicumyl peroxide (DCP), can improve efficiency and reduce time and temperature requirements [12, 13]. The vulcanization accelerator interacts with the active agent in the vulcanization system under heating conditions, so as to promote the ring-opening reactions of sulfur molecules, accelerating the crosslinking speed of rubber molecular chains, to form a three-dimensional network structure fast, which is the essential step for NRLF with high elasticity [14–16].

DPG is an important industrial chemical, which is widely used in rubber industry, biology, medicine, and other fields [17]. DPG is often used as a secondary accelerator in the vulcanization process, endowing the rubber with high modulus and high tensile strength. Because DPG is difficult to dissolve and disperse in water, rubber industry generally needs ball milling to maintain the dispersion effect of DPG; otherwise, DPG would make NR latex exhibit gelation, casting a negative effect on the vulcanization process of rubber [18]. In addition, exposure to DPG during production process or in direct contact with the final products may cause allergic contact dermatitis, reproductive toxicology, cytostatic and mutagenicity, and environmental pollution [19]. These problems make the application of DPG greatly restricted.

Cyclodextrins are compounds derived from the enzymatic degradation of starch, one of the most important polysaccharides, which are macrocyclic molecules [20, 21]. The core of their structure consists of a hydrophobic cavity with stable size that can trap or encapsulate other molecules [22–25]. The encapsulation property of the host-guest relationship can improve the physical, chemical, and biological properties of the guest molecules [26, 27]. *β*-Cyclodextrin (*β*-CD) is a cyclic oligosaccharide composed of glucuronic acid monomers linked by *α*-(1,4) bond, which has the hydrophobic interior and hydrophilic exterior [28]. This structure can cover lipophilic molecules with appropriate size and form cyclodextrin-guest molecular inclusion complex, which can effectively improve the properties of low solubility compounds [29–31]. Hydroxypropyl-*β*-cyclodextrin (HP-*β*-CD) is the most used derivative of *β*-CD and is synthesized by substituting hydroxypropyl groups for the -OH group on the surface of *β*-CD. Compared with *β*-CD, HP-*β*-CD has better water solubility (>600 mg/mL) and inclusion ability, as well as lower toxicity [32].

To tackle the limitations of DPG in the vulcanization process, we have prepared the inclusion complex of diphenylguanidine-hydroxypropyl-*β*-cyclodextrin (DPG-HP-*β*-CD) by ball milling. The water solubility and dispersibility of DPG are improved through host-guest interaction. The inclusion complex can keep NR latex stable without gelling before vulcanization. During the vulcanization, the temperature reaches about 100°C, the stability of the inclusion complex is affected by the external environment, and the dissociation of the host and guest molecules occurs spontaneously. However, due to the full dispersion of the inclusion complex before vulcanization in the system, it is conducive to the efficient reaction of DPG in the vulcanization reaction, so as to inhibit the phenomenon of partial overvulcanization or incomplete reaction. We have confirmed that the host-guest molar ratio of the inclusion complex is 1 : 1 by the Boltzmann-Hamel method, molecular docking simulation, and phase solubility method. Meanwhile, the solubilization coefficient KC of HP-*β*-CD to DPG has also measured to be 152 L/mol and the solubility of DPG increased linearly with the increase of HP-*β*-CD concentration. According to the solubility experiment, the concentration of DPG in the saturated DPG-HP-*β*-CD aqueous solution is calculated to be 0.064 mmol/L, which is 14.5 times higher than that of pure DPG, indicating that the solubilization effect of HP-*β*-CD on DPG is obvious. In addition, we applied the highly water-soluble DPG-HP-*β*-CD inclusion complex to the foaming process of NRLF, comparing the tensile strength, elongation at break, hardness, compression set, aging, and resilience. DPG-HP-*β*-CD/NRLF has obvious improvement both in mechanical properties, antiaging performance, and biocompatibility.

## 2. Result and Discussion

### 2.1. Research on DPG-HP-*β*-CD

**Figure 1:**
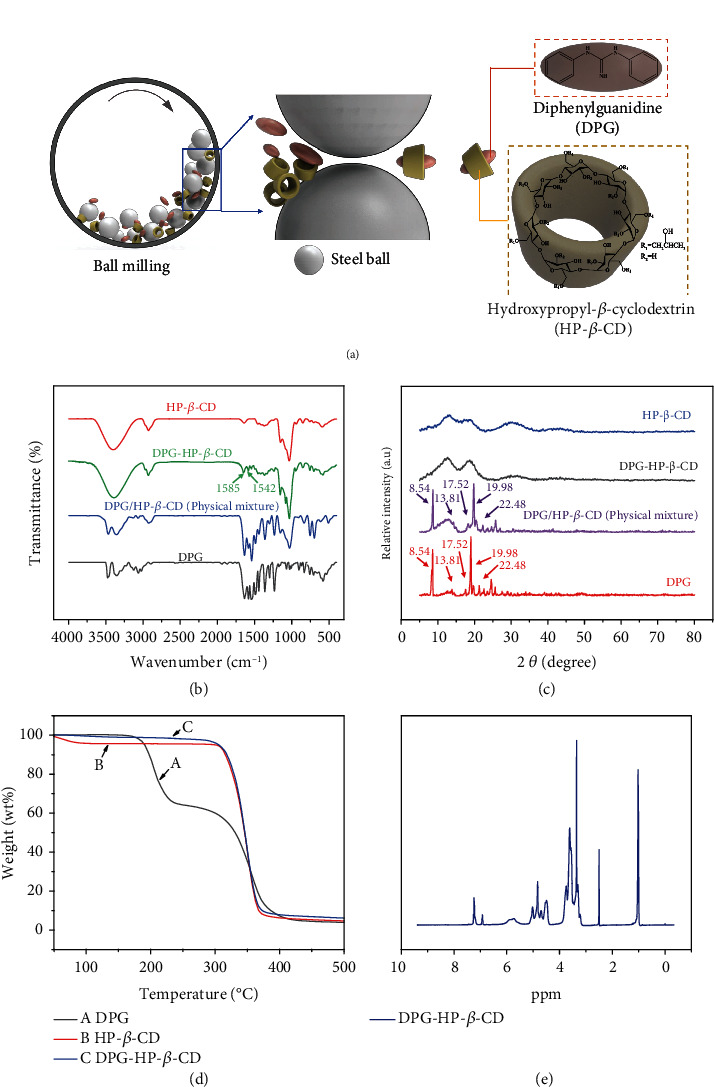
Preparation and characterization of the inclusion complex. (a) Schematic diagram of the inclusion complex of DPG-HP-*β*-CD through ball milling. (b) FT-IR, (c) XRD patterns, and (d) TGA of DPG-HP-*β*-CD and its comparisons. (e) ^1^H NMR spectra of DPG-HP-*β*-CD.

There are many methods to prepare cyclodextrin inclusion complex, which can be divided into solution method and solid phase method. Additionally, the solid phase method includes grinding and ball milling. The DPG-HP-*β*-CD is prepared by ball milling, which is usually used for mass production (Figure 1(a)). As shown in Table S1, the encapsulation efficiency increases at the first 40 min and then decreases with the increase of ball milling time, which finally stabilized at 22%. The encapsulation efficiency reaches the maximum 30% at 60 min. Due to the dynamic process of the encapsulation between DPG and HP-*β*-CD, longer milling time may offer more chances for DPG to enter the HP-*β*-CD cavity, but the encapsulations between host and guest finally achieve the dynamic equilibrium state with the extension of ball milling time [33, 34].

As shown in Figure 1(b), FT-IR spectra reveal functional groups and intermolecular interactions between host and guest. The peaks at 3473 cm^−1^ and 3352 cm^−1^ are the deformation vibration peaks of N-H in DPG. Further, the peaks at 1634 cm^−1^, 1581 cm^−1^, 1540 cm^−1^, and 1442 cm^−1^ are attributed to the stretching vibration peaks of benzene ring in DPG [35]. For the HP-*β*-CD, the peaks that occurred at 3400 cm^−1^, 2925 cm^−1^, 1637 cm^−1^, 1151 cm^−1^, and 1033 cm^−1^ correspond to the stretching vibration peaks of C-H and -CH_2_, deformation vibration peak of H-O-H, and stretching vibration peaks of C-O and C-O-C, respectively. From the FT-IR spectra of DPG-HP-*β*-CD, it has obvious characteristic peaks both of DPG and HP-*β*-CD, indicating that there exists no interaction between the mixtures which are physically mixed [36]. Due to the shielding effect of the cavity, DPG-HP-*β*-CD loses the characteristic peaks within 1000-1450 cm^−1^ and 3000-3500 cm^−1^, which belong to DPG. However, the characteristic peaks of HP-*β*-CD are retained. Additionally, we could find weak deformation vibration of the benzene ring skeleton of DPG, indicating that the vibration of benzene ring skeleton is limited, and the two peaks have slight red shift. Eventually, this phenomenon proves that there has host-guest interactions between DPG and HP-*β*-CD, thus encapsulating DPG into the cavity of HP-*β*-CD. As shown in Figure 1(c), the crystal structures of DPG-HP-*β*-CD and its comparisons are analyzed by XRD. DPG has good crystallinity with sharp diffraction peaks at 8.54°, 13.81°, 17.52°, 18.98°, and 22.48°. As a comparison, the diffraction pattern of HP-*β*-CD has no obvious crystal peak, which reflects that HP-*β*-CD exists in an amorphous state [37, 38]. In the diffraction pattern of the physical mixture of DPG and HP-*β*-CD, there are both the amorphous state of HP-*β*-CD and multiple diffraction peaks of DPG, indicating that no new crystals are formed between the physical mixtures. Compared with DPG and HP-*β*-CD, the diffraction pattern of DPG-HP-*β*-CD inclusion complex is similar to HP-*β*-CD, in which the characteristic peaks of DPG nearly disappear. Because DPG is encapsulated into the cavity of HP-*β*-CD, the crystal characteristics of DPG disappear, which further verifies the successful formation of the DPG-HP-*β*-CD inclusion complex. As shown in Figure 1(d), the thermal stability and components of DPG, HP-*β*-CD, and DPG-HP-*β*-CD are evaluated by thermogravimetric analysis (TGA). The thermal decomposition process of DPG has gone through two stages. The first stage is between 166 and 256°C, which is the melting of DPG, and the second stage is between 256 and 500°C, which is the decomposition of DPG. In addition, HP-*β*-CD has two stages, namely, the dehydration (30-100°C) and the decomposition of macrocyclic molecules (302-500°C). The dehydration stage of the decomposition process of DPG-HP-*β*-CD inclusion complex almost disappears because DPG molecules replace high-energy water molecules encapsulated in HP-*β*-CD cavities. By analyzing the integral area of the H proton peak in the ^1^H NMR spectrum, the molar ratio of the inclusion complex formed by DPG and HP-*β*-CD can be obtained. Furthermore, the chemical shift (*δ*) could be used to check whether the inclusion complex is successfully formed. As it is a noncovalent interaction between DPG and HP-*β*-CD, the change in chemical shift is relatively small. Figure 1(e) and Figures S1a and S1b are the ^1^H NMR spectra of DPG-HP-*β*-CD, DPG, and HP-*β*-CD, respectively. In terms of the DPG-HP-*β*-CD inclusion complex, the intensities of the characteristic peaks at 6.88 ppm and 7.22 ppm of DPG are greatly weakened, due to the host-guest interaction between DPG and HP-*β*-CD. HP-*β*-CD produces a shielding effect on DPG, which indicates the successful formation of DPG-HP-*β*-CD inclusion complex. Compared with the ^1^H NMR spectra of DPG, the H-a and H-b, c of DPG in DPG-HP-*β*-CD move to the downfield from 6.88 ppm and 7.22 ppm to 6.92 ppm and 7.24 ppm, respectively. Moreover, H-1 and H-5,6 of HP-*β*-CD also shift to downfield, which reveal the result of the host-guest interaction between DPG and HP-*β*-CD (Table S2) [39]. By calculating the integrated peak ratio of HP-*β*-CD and DPG, their molar ratio can be obtained, as shown below:

(1) $$\begin{array}{c} \text{HP}‐\beta ‐\text{CD intensity}=1.00\;\left(7\text{ protons}\right),\\ DPG\;intensity=0.20\left(2\;protons\right),\\ \text{Ratio of intensity}=\frac{2/7}{0.2/1}=1.42. \end{array}$$

Therefore, the molar ratio of DPG to HP-*β*-CD is calculated to be approximately 1 : 1 based on the ^1^H NMR spectra.

**Figure 2:**
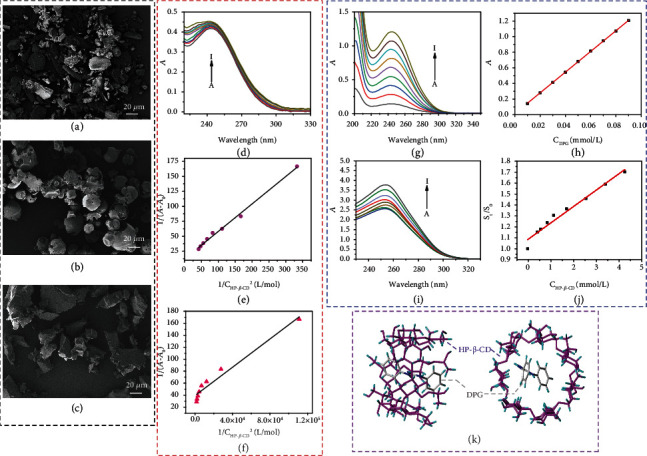
Molar ratio of the host-guest inclusion complex. SEM images of (a) DPG, (b) HP-*β*-CD, and (c) DPG-HP-*β*-CD inclusion complex. (d) UV spectra of 3 × 10^−5^ mol/L DPG (ethylene glycol) solution and HP-*β*-CD (ethylene glycol) solution with different concentrations (10^−3^ mol/L) at 25°C: 0, 3, 6, 9, 12, 15, 18, 21, and 24. (e) Linear fitting curve of 1/[*A* − *A*_0_] to 1/[*H*] and (f) linear fitting curve of 1/[*A* − *A*_0_] to 1/[*H*]^2^. (g) UV spectra of DPG (ethylene glycol) solution with gradient concentrations (mmol/L) at 25°C: 0.01, 0.02, 0.03, 0.04, 0.05, 0.06, 0.07, 0.08, and 0.09. (h) Linear fitting curve of the absorbance at 244 nm of DPG (ethylene glycol) solution to the actual concentration. (i) UV spectra of excess DPG aqueous solution and HP-*β*-CD (ethylene glycol) solution with different concentrations (mmol/L): 0, 0.43, 0.57, 0.86, 1.14, 1.71, 2.56, 3.42, and 4.28. (j) Dissolution curve of DPG in HP-*β*-CD aqueous solution. (k) Docking modeling of HP-*β*-CD and DPG: left: side view of DPG-HP-*β*-CD inclusion complex; right: top view of DPG-HP-*β*-CD inclusion complex.

As shown in Figures 2(a) and 2(b), DPG is a rod-like crystal with an irregular surface, while the surface morphology of HP-*β*-CD is spherical with a cavity inside. From the SEM image of DPG-HP-*β*-CD inclusion complex (Figure 2(c)), the rod-like structures disappear, and the cavities of HP-*β*-CD are filled with DPG, showing block-like structures of irregular sizes. The supramolecular interactions between DPG and HP-*β*-CD result in the morphological changes.

Furthermore, UV spectra are an effective tool for estimating host-guest interactions of inclusion complex, which detects the increase or decrease of the absorption intensity of the solution with different concentrations of host and guest. As shown in Figure 2(d), the absorbance of DPG increases linearly with the increase of HP-*β*-CD concentration, which indicates the existence of host-guest interactions between DPG and HP-*β*-CD. Assuming that the molar ratio of DPG to HP-*β*-CD is 1 : 1 or 1 : 2, the following equations are used for calculation [40, 41].

(2) $$\begin{array}{c} \frac{1}{\left[A-A_{0}\right]}=\frac{1}{\left[\Delta \varepsilon \right]}+\frac{1}{K_{a}{\left[G\right]}_{0}\left[\Delta \varepsilon \right]\left[H\right]}, \end{array}$$

(3) $$\begin{array}{c} \frac{1}{\left[A-A_{0}\right]}=\frac{1}{\left[\Delta \varepsilon \right]}+\frac{1}{K_{a}{\left[G\right]}_{0}\left[\Delta \varepsilon \right]{\left[H\right]}^{2}}, \end{array}$$

(4) $$\begin{array}{c} \Delta \varepsilon =\varepsilon_{HG}-\varepsilon_{H}-\varepsilon_{G}. \end{array}$$

[*G*]_0_ is the initial concentration of guest molecule (DPG), mol/L; [*H*] is the concentration of host molecule (HP-*β*-CD), mol/L; *A* is the absorbance of DPG after adding HP-*β*-CD; *A*_0_ is the absorbance of pure DPG solution; [*A* − *A*_0_] is the absorbance change (the absorbance of the inclusion complex); *ε*_*H*_, *ε*_*G*_, and *ε*_*HG*_ are the molar absorption coefficients of *H*, *G*, and *HG*, respectively; *K*_*a*_ is the binding constant of host and guest molecules, L/mol.

According to Equations (2) and (3), the linear fitting curves illustrate the phenomenon. When the molar ratio of DPG and HP-*β*-CD is assumed to be 1 : 1, the linear correlation is better (*R*^2^ = 0.995). When the molar ratio is assumed to be 2 : 1, the linear correlation is only 0.955 (Figures 2(e) and 2(f)). This phenomenon further confirms the molar ratio of DPG-HP-*β*-CD inclusion complex is about 1 : 1. According to Equation (4), *K*_*a*_ of DPG-HP-*β*-CD is calculated to be 8.621 × 10^5^ L/mol.

The absorbance of DPG increases with the increase of concentration, and they have a good linear correlation (*R*^2^ = 0.999), as shown in Figures 2(g) and 2(h). We can calculate the molar absorption coefficient of DPG to be 4.43 L/(mmol·cm) according to the Lambert-Beer equation. Additionally, HP-*β*-CD has no UV absorption peak in the range of 220-400 nm which would not affect the inclusion complex, while DPG and DPG-HP-*β*-CD inclusion complex have obvious absorption peaks at 244 nm (Figure S2). The solubility of DPG is significantly improved by the inclusion of HP-*β*-CD. According to the Lambert-Beer equation, we could calculate that the solubility of DPG-HP-*β*-CD inclusion complex is 0.064 mmol/L, while that of DPG is only 0.0044 mmol/L, which has an increase of 14.5 times. The solubilization effect of DPG under different concentrations of HP-*β*-CD is illustrated in Figures 2(i) and 2(j), and the solubility of DPG in water increases with the increase of the concentration of HP-*β*-CD. Its linear relationship can be expressed by the following equation:

(5) $$\begin{array}{c} \frac{S_{t}}{S_{0}}=1+K_{C}C_{0}. \end{array}$$

*S*_*t*_ represents the concentration of DPG in solutions with different concentrations of HP-*β*-CD, mmol/L; *S*_0_ represents the intrinsic concentration of DPG, mmol/L; *C*_0_ represents the initial concentration of HP-*β*-CD, mmol/L; *K*_*C*_ represents solubilization coefficient, L/mol, which is used to evaluate the solubilizing ability of HP-*β*-CD to DPG.

According to Equation (5), *K*_*C*_ of HP-*β*-CD to DPG can be calculated as 152 L/mol, indicating that HP-*β*-CD has a certain solubilization effect on DPG. In addition, the superior linear correlation result (*R*^2^ = 0.978) once again assists to prove that DPG and HP-*β*-CD are 1 : 1 molar ratio inclusion complex. The optimal inclusion state of HP-*β*-CD and DPG is revealed by molecular simulation (Figure 2(k)). The results illustrate that DPG could enter the cavity of HP-*β*-CD with a stable state after inclusion, where one DPG molecule only enters one HP-*β*-CD cavity.

### 2.2. Research on DPG-HP-*β*-CD/NRLF

**Figure 3:**
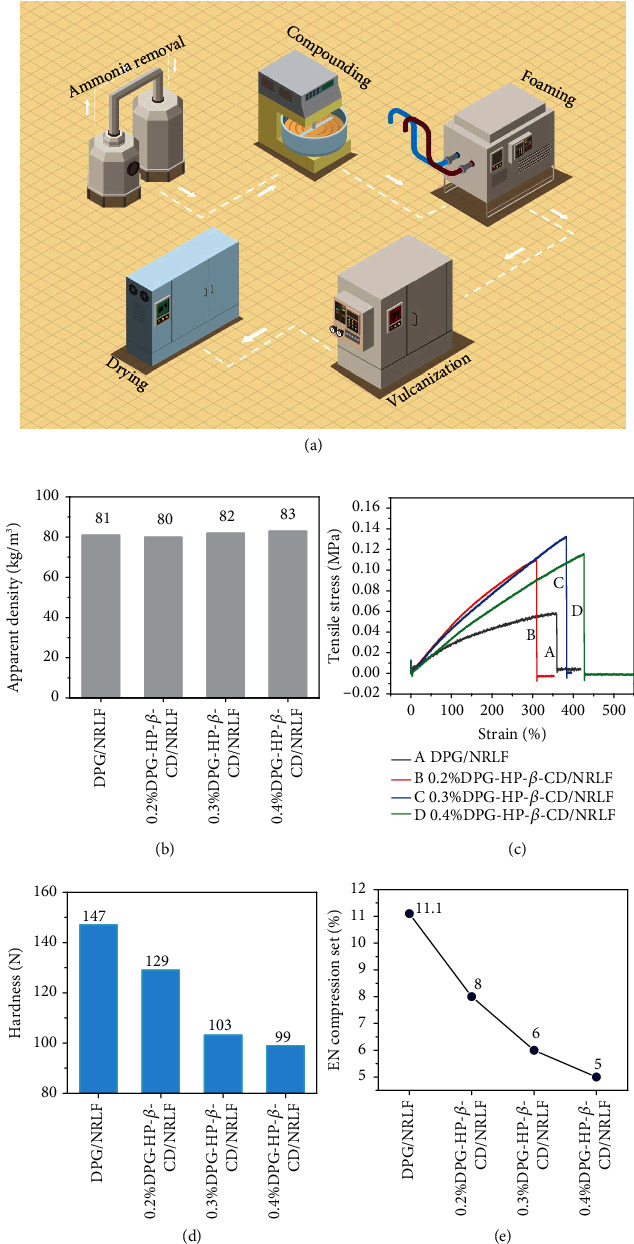
The changing trend of mechanical properties during industry production. (a) Industrial production flowchart of natural rubber latex foam. (b) Apparent density. (c) Typical stress-strain curve, (d) hardness, and (e) EN compression set of DPG/NRLF, 0.2%, 0.3%, and 0.4%DPG-HP-*β*-CD/NRLF.

Vulcanization is an important stage in the process of NR production. And vulcanization accelerator can promote the crosslinking reactions of rubber molecular chains, which improve the vulcanization speed to reduce the request of time and temperature. In industry, the Dunlop intermittent foaming method is often used to prepare NRLF, as shown in Figure 3(a). The NR latex should be first stirred to remove ammonia. After that, the sulfur dispersion, potassium pyrophosphate, potassium oleate, castor oil, potassium hydroxide, and DPG need to be added into the NR latex and stirred at 38°C for 4 h. The NR latex mixture is transferred to the foaming machine for mechanical foaming. Zinc oxide and sodium fluorosilicate dispersion are added during the foaming process, and the foam is injected into the mold immediately as soon as the foam is initiated. Finally, the mold should be placed in a steam oven at 100°C for vulcanization and shaping to obtain NRLF.

From the comparison of apparent densities in Figure 3(b), the density of DPG/NRLF is 81 kg/m^3^, and the density of DPG-HP-*β*-CD/NRLF is not much different from that of DPG/NRLF, which slightly increased with the increase of DPG-HP-*β*-CD content. Since the density of NRLF is related to the foaming process, it shows that the foaming degrees of all samples in the experiment are almost the same, which is the premise of the mechanical tests. From Figure 3(c), the NRLF only have an elastic deformation stage during the stretching, without any yield stage, strengthening stage, or necking stage [42]. The tensile strength of DPG-HP-*β*-CD/NRLF is significantly better than that of DPG/NRLF. With the increase of DPG-HP-*β*-CD content, the tensile strength of NRLF first increased and then decreased, but it remains at 84 kPa in the end, which is about 1.2 times higher than that of DPG/NRLF. However, the tensile strength of 0.4%DPG-HP-*β*-CD/NRLF does not further improve, because the promotion effect of DPG has reached the upper limit. If there is too much DPG, overvulcanization would occur, which could reduce the tensile strength of the NRLF productions. To sum up, the DPG-HP-*β*-CD inclusion complex can greatly enhance the utilization rate of DPG in NRLF and improve the tensile strength of NRLF. Table S3 illustrates that the rebound rate and rebound height of DPG-HP-*β*-CD/NRLF fluctuate around 61% and 280 mm, which are a little higher than DPG/NRLF. As shown in Figure 3(d), the hardness of DPG/NRLF is as high as 147 N while the hardness of DPG-HP-*β*-CD/NRLF decreases significantly. NRLF gradually become softer with the increase of DPG-HP-*β*-CD content. Since the crosslinking degree of DPG/NRLF is not enough, the porous structure of foam is limited, resulting in the relatively high hardness. As a comparison, DPG-HP-*β*-CD/NRLF has a higher degree of crosslinking, and the hardness decreases accordingly, which is consistent with the tendency of tensile strength. Furthermore, the EN compression set of DPG/NRLF is 11.1%, while that of DPG-HP-*β*-CD/NRLF is smaller (Figure 3(e)). With the increase of DPG-HP-*β*-CD content, the EN compression set also decreases, which indicates that DPG-HP-*β*-CD improves the mechanical performance.

**Figure 4:**
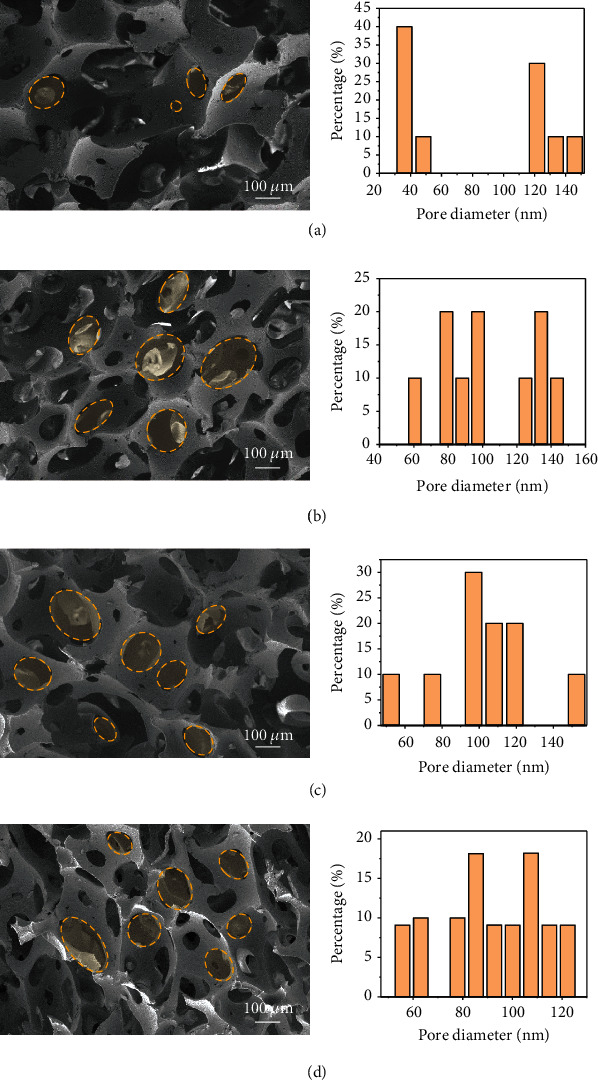
Changes in the porous structure of NRLF. SEM images of DPG-HP-*β*-CD/NRLF with corresponding pore diameter distribution: (a) DPG/NRLF, (b) 0.2%DPG-HP-*β*-CD/NRLF, (c) 0.3%DPG-HP-*β*-CD/NRLF, and (d) 0.4%DPG-HP-*β*-CD/NRLF.

DPG/NRLF have low porosity and small pore size with low uniformity (Figure 4(a)), while DPG-HP-*β*-CD/NRLF have a significant enhancement in porosity and the pore size are more uniform. With the increase of DPG-HP-*β*-CD content (from 0.2% to 0.4%), the morphologies of latex foams do not change obviously (Figures 4(b)–4(d)). The size, uniformity, and porosity of the NRLF pores are related to the gelation process of the latex foam. The quality of the porous structure of the latex foam depends on whether the interfacial free energies between the whey-rubber and whey-air interface of the ungelled latex foam can be well controlled. Due to the high water-soluble DPG-HP-*β*-CD which can increase the free energy of whey-rubber interface fast, DPG-HP-*β*-CD/NRLF have the promising porosity. Further, Table S4 illustrates the relevant data measured by NMR, where *T*_2_ is the transverse relaxation time of hydrogen proton in the magnetic field, reflecting the high frequency and fast movement of rubber molecular chains, such as the movement of uncrosslinked molecules, small molecules, and terminal free chains. 0.3%DPG-HP-*β*-CD/NRLF has the highest proportion of catenary (39.01%), and therefore, *T*_2_ has the highest proportion. As a comparison, DPG/NRLF has the lowest proportion of catenary (27.07%), and *T*_2_ is also short. *M*_*C*_ is the relative molecular mass between crosslinking points, which can be calculated by the following formula:

(6) $$\begin{array}{c} M_{c}=\frac{3CM_{r}}{5n\sqrt{qMrl}}. \end{array}$$

*C* is the number of bonds/statistical chain segments of the main chain in the repeating unit, 7.2 for natural rubber; *M*_*r*_/*n* is the molar mass of the monomer unit/the number of bonds in the main chain, which is 68/4 for natural rubber; *qMrl* is the remaining dipole moment.

As shown in Figure S3, the crosslinking density of DPG/NRLF, 0.2%, 0.3%, and 0.4%DPG-HP-*β*-CD/NRLF shows an opposite trend to *M*_*C*_. With the increase of DPG-HP-*β*-CD content, the crosslinking density of rubber increases first and then decreases, while *M*_*C*_ decreases first and then increases. The increase of crosslinking density indicates that the number of crosslinking points of rubber molecules increases, and the rubber molecular chain between adjacent crosslinking points becomes shorter. The relative molecular mass between rubber crosslinking points becomes smaller.

**Figure 5:**
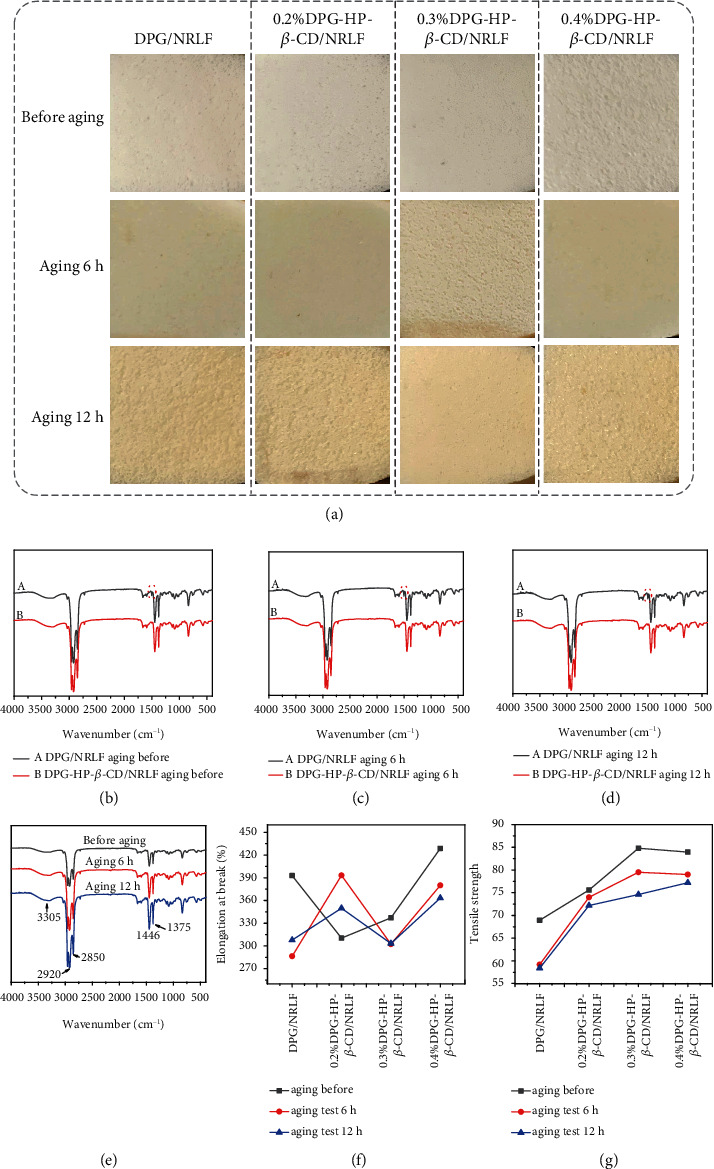
Research on antiaging properties of DPG-HP-*β*-CD/NRLF. (a) Digital photos of DPG/NRLF, 0.2%, 0.3%, and 0.4%DPG-HP-*β*-CD/NRLF before aging, after aging 6 h, and aging 12 h at 100°C. FT-IR spectra of DPG/NRLF and DPG-HP-*β*-CD/NRLF with the conditions of (b) aging before, (c) aging for 6 h, and (d) aging for 12 h and (e) the comparison of DPG-HP-*β*-CD/NRLF within different conditions. (f) Tensile strength and (g) elongation at break of DPG/NRLF, 0.2%, 0.3%, and 0.4%DPG-HP-*β*-CD/NRLF before aging, after aging 6 h, and aging 12 h.

NRLF products are easily influenced by external environmental factors during processing, storage, and use, which would react with nitrogen oxides and O_2_ in the air to form colored groups (aging phenomenon), leading to the surface turning yellow and performance degradation. We accelerate the aging of DPG/NRLF, 0.2%, 0.3%, and 0.4%DPG-HP-*β*-CD/NRLF for 6 h and 12 h, respectively, as shown in Figure 5(a). In the digital photos of the four groups of NRLF samples before aging and 6 h and 12 h aging, it can be found that there exist small changes in color between 6 h aging and before aging. However, it could be felt that the surface of the NRLF has been oxidized by touching. After aging for 12 hours, the obvious oxide layer appears on the NRLF, and the surfaces begins to turn yellow, especially for the yellowing of DPG/NRLF with the tendency to develop inward. As illustrated in the SEM images of Figures S4a–S4h, four groups of NRLF samples do not change significantly after 6 h of aging, which are compared with that before aging. It is evident that after 12 h of aging, the two groups of DPG/NRLF and 0.2%DPG-HP-*β*-CD/NRLF exhibit obvious cracks (Figures S4i and S4j). However, this phenomenon does not occur in the 0.3% and 0.4%DPG-HP-*β*-CD/NRLF groups (Figures S4k and S4l). Digital photographs and SEM images can only provide a macro understanding, so analyzing changes in functional groups will shed more light on the aging process. In the FTIR spectra of DPG/NRLF, there is an obvious characteristic peak at 1600 cm^−1^, which corresponds to the stretching vibration of C=O in -COOH, and this peak becomes more and more obvious as the aging time increases (Figures 5(b)–5(d)). It is worth noting that it is an enhancement of the absorptions that were being looked for and not simply their presence or absence [43]. This phenomenon implies that aerobic functional groups are formed in DPG/NRLF during the aging process, but this phenomenon does not exist in the FTIR spectra of DPG-HP-*β*-CD/NRLF during the aging process. As shown in Figure 5(e), the comparison of FTIR spectra of DPG-HP-*β*-CD/NRLF before and after aging shows no significant change, indicating that only a small part of the NRLF is oxidized after 6 h and 12 h aging. The elongation at break of NRLF showed an upward trend in the aging process (Figure 5(f)). Due to the crosslinked structures that are not conducive to the stretching of molecular chains, aging process would destroy the network structure of NRLF so that some entangled rubber molecular chains are released and oriented in the direction that is conducive to the stretching of molecular chains. As illustrated in Figure 5(g), the tensile strength of DPG/NRLF decreases by 14.1% after aging for 6 h, and the final tensile strength decreases by 15.3% after aging for 12 h. However, the tensile strength of 0.2%, 0.3%, and 0.4%DPG-HP-*β*-CD/NRLF decreases by 2.1%, 6.3%, and 5.9% after aging for 6 h, and the total tensile strength decreases by 4.5%, 12%, and 8% after aging for 12 h, respectively. The tensile strength of NRLF decreases significantly after 6 h aging, while the decreasing tendency after the continued 6 h aging becomes smaller, which indicates that the aging rate in the early stage is greater than that in the later stage. Additionally, the decrease of the tensile strength of DPG-HP-*β*-CD/NRLF is significantly smaller than that of DPG/NRLF, indicating the antiaging performance of DPG-HP-*β*-CD/NRLF. After 12 h of aging, numerous cracks and holes appear on the surface structure of DPG/NRLF, which causes the mechanical properties weakening (Figure S5a). In comparison, NRLF with DPG-HP-*β*-CD have almost no damage (Figures S5b–S5d).

**Figure 6:**
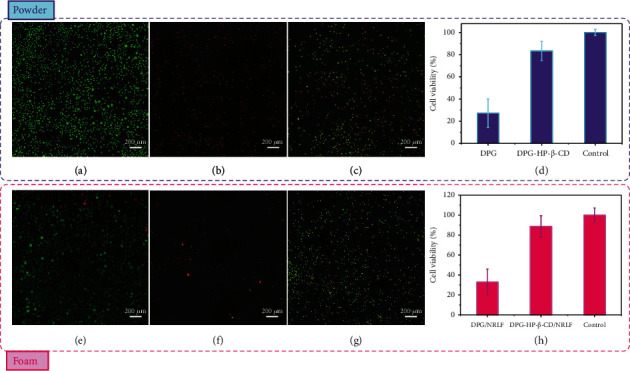
The improved biocompatibility of DPG-HP-*β*-CD/NRLF. The in vitro cytotoxicity results: microscopic images of L929 cells cultured for 24 h in (a) medium (control), (b) extract substrate of DPG, and (c) extract substrate of DPG-HP-*β*-CD. (d) Optical density of L929 cells cultured for 24 h in medium, extract substrate of DPG, and extract substrate of DPG-HP-*β*-CD; microscopic images of L929 cells cultured for 24 h in (e) medium (control), (f) extract substrate of DPG/NRLF, and (g) extract substrate of DPG-HP-*β*-CD/NRLF. (h) Optical density of L929 cells cultured for 24 h in medium, extract substrate of DPG/NRLF, and extract substrate of DPG-HP-*β*-CD/NRLF.

To conduct the toxicity of DPG-HP-*β*-CD and DPG-HP-*β*-CD/NRLF, the in vitro cytotoxicity test is performed by using L929 cells cultured with extract substrates for 24 h. As shown in Figures 6(a) and 6(e), the L929 cells cultured for 24 h in medium displayed the fusiform morphology. When L929 cells are cultured for 24 h in the extract substrate of DPG, lots of cells have died, and the cell viability is only 27.23% (Figures 6(b) and 6(d)). Surprisingly, the extract substrate of DPG-HP-*β*-CD inclusion complex has obvious low cytotoxicity, and L929 cells can survive stably with a viability of 88.69% (Figures 6(c) and 6(d)). The cytotoxicity of DPG/NRLF and DPG-HP-*β*-CD/NRLF is similar to that of monomer powder, as shown in Figures 6(f) and 6(g). The cell viabilities are 32.91% and 88.69% for the extract substrates of DPG/NRLF and DPG-HP-*β*-CD/NRLF, respectively (Figure 6(h)). Overall, DPG-HP-*β*-CD has lower cytotoxicity compared to DPG, due to the host-guest structure of the inclusion complex, which restricts the efflux of toxicants within the DPG. DPG-HP-*β*-CD/NRLF has good biocompatibility and is promising for environmental-friendly and bio-friendly industrial NRLF production.

## 3. Conclusions

The purpose of this paper is to solve the insoluble and toxic problems of DPG when it is used as a vulcanization accelerator. Hence, we innovatively propose a design strategy for the inclusion complex of DPG-HP-*β*-CD by ball milling. Through host-guest interactions, HP-*β*-CD endows the DPG with greatly enhanced water solubility and dispersibility. We have confirmed that the host-guest molar ratio of the DPG-HP-*β*-CD inclusion complex is 1 : 1. The DPG-HP-*β*-CD inclusion complex is used to the foaming process of NRLF; the antiaging ability and the mechanical properties of the final obtained DPG-HP-*β*-CD/NRLF are significantly improved. Either DPG-HP-*β*-CD or DPG-HP-*β*-CD/NRLF, their biocompatibility is much better than DPG-only products. The successful development of DPG-HP-*β*-CD will greatly reduce the harm to the environment and human body in the industrial production of NR latex. It is a promising product with high potential for the vulcanization secondary accelerator in the future.

## 4. Materials and Methods

### 4.1. Materials

1,3-Diphenylguanidine, potassium bromide, deuterated dimethyl sulfoxide, potassium pyrophosphate, potassium oleate, potassium hydroxide, ammonia, sodium fluorosilicate, formaldehyde, ethylene glycol, and ethanol are purchased from Sinopharm Chemical Reagent Co., Ltd. Hydroxypropyl-*β*-cyclodextrin is purchased from Aladdin Reagent Shanghai Co., Ltd.

### 4.2. Characterization

The polarizing microscope (Nikon ECLIPSE, LV100) and the field emission scanning electron microscope (FESEM, Hitachi S-4800) are used to characterize the microstructure of samples. The crystal structures of DPG-HP-*β*-CD and its comparisons are determined by an X-ray diffractometer (XRD, Bruker D8 Advance with Cu K*α* radiation *λ* = 0.15406 nm). Fourier transform infrared (FT-IR) spectroscopy (Alpha, Bruker, Karlsruhe, Germany) verified the physicochemical composition of the samples. Thermogravimetric analysis (TGA, Mettler Toledo) is used to determine the degree of thermal decomposition and component content. The ^1^H NMR spectra are conducted on a 600 MHz Bruker spectrometer (Bruker, Germany) at 303.1 K in DMSO. An Instron Mechanical Tester (model 3367) is used to determine the mechanical properties of the samples under the test rate of 50 mm·min^−1^.

### 4.3. Preparation of DPG-HP-*β*-CD Inclusion Complex

DPG-HP-*β*-CD inclusion complex is prepared by ball milling. The ball-milling process was performed in 15 mL hardened stainless steel jar with one stainless steel ball of mass 4.0 g. The filling ratio of the steel balls is 40%. Weigh 0.1 mol/L HP-*β*-CD and 0.1 mol/L DPG in the ball-milling jar, respectively, and ball mill at 21.00 Hz for 20, 40, 60, 80, and 100 min, respectively. Unreacted DPG is filtered off, and the filtrate is rotary-evaporated and vacuum-dried for 24 h.

### 4.4. Host-Guest Interaction of DPG-HP-*β*-CD Inclusion Complex

According to the Benesi-Hildebrand (B-H) method, the molar ratio of DPG and HP-*β*-CD inclusion complex is determined by a UV spectrophotometer.

### 4.5. Molecular Simulation of DPG-HP-*β*-CD Inclusion Complex

Download the crystal structure file of *β*-CD from the Crystallography Open Database, and then, use Gaussian 09 software to replace the 2-hydroxyl group in the *β*-CD model with 2-hydroxypropyl. After optimization, the HP-*β*-CD model can be constructed. ChemOffice software is used to build the molecular model of DPG. We use Chem3D to obtain the minimum energy conformation of the molecule. Finally, molecular docking is performed by SYBYL-X 2.0 software.

### 4.6. Determination of Apparent Density

According to GB9891-1988 determination of apparent density of latex foam, the apparent density of the samples is calculated by the following equation:

(7) $$\begin{array}{c} \rho_{a}=\frac{m}{V}\times 10^{6}. \end{array}$$

*ρ*_*a*_ is the apparent density, kg/m^3^; *m* is the sample mass, g; *V* is the sample volume, mm^3^.

### 4.7. Determination of Hardness

The hardness of the NRLF samples is tested under the test conditions of the B method (H-100) with GB10807-2006 as the reference standard.

### 4.8. Determination of Resilience

The ball rebound tester (MCGS) is used to test the resilience of the samples according to the GB/T1.1-2009 standard.

### 4.9. Determination of EN Compression Set

The samples are compressed to 75% of their original thickness and placed at 70 ± 1°C for 22 h. The thickness is measured according to the provisions.

### 4.10. Determination of Crosslink Density

The NRLF samples are measured by nuclear magnetic resonance (NMR) spectroscopy (VTMR20-010V-1, Suzhou Niumag Corporation, China). The test is performed at 0.5 ± 0.05 T magnetic induction intensity, 15 MHz, and 90°C.

## Data Availability

All other data are available from the corresponding authors upon reasonable request.

## Supplementary Materials

Supplementary Materials Supplementary Materials Figure S1: ^1^H NMR spectra of (a) DPG and (b) HP-*β*-CD. Figure S2: UV spectra of HP-*β*-CD, DPG, and DPG-HP-*β*-CD. Figure S3: crosslinking density and relative molecular mass between crosslinking points. Figure S4: SEM images of DPG/NRLF, 0.2%, 0.3%, and 0.4%DPG-HP-*β*-CD/NRLF before aging (a–d), after aging 6 h (e–h), and aging 12 h at 100°C (i–l). Figure S5: SEM images of (a) DPG/NRLF, (b) 0.2%, (c) 0.3%, and (d) 0.4%DPG-HP-*β*-CD/NRLF after aging 12 h at 100°C. Table S1: contents of DPG in DPG-HP-*β*-CD inclusion complex under different ball milling times. Table S2: chemical shift of H protons of DPG-HP-*β*-CD, HP-*β*-CD, and DPG. Table S3: the rebound rate and rebound height of DPG/NRLF, 0.2%, 0.3%, and 0.4%DPG-HP-*β*-CD/NRLF. Table S4: DPG/NRLF, 0.2%, 0.3%, and 0.4%DPG-HP-*β*-CD/NRLF crosslinking density results.
