# Supplementary material for: Design Strategy for Vulcanization Accelerator of Diphenylguanidine/Cyclodextrin Inclusion Complex for Natural Rubber Latex Foam with Enhancing Performance
Source: Research (Wash D C). 2022 Sep 1;2022:9814638. doi: 10.34133/2022/9814638 (PMC9470207; doi:10.34133/2022/9814638)
Supplement: Supplementary Materials — Figure S1: 1H NMR spectra of (a) DPG and (b) HP-β-CD. Figure S2: UV spectra of HP-β-CD, DPG, and DPG-HP-β-CD. Figure S3: crosslinking density and relative molecular mass between crosslinking points. Figure S4: SEM images of DPG/NRLF, 0.2%, 0.3%, and 0.4%DPG-HP-β-CD/NRLF before aging (a–d), after aging 6 h (e–h), and aging 12 h at 100°C (i–l). Figure S5: SEM images of (a) DPG/NRLF, (b) 0.2%, (c) 0.3%, and (d) 0.4%DPG-HP-β-CD/NRLF after aging 12 h at 100°C. Table S1: contents of DPG in DPG-HP-β-CD inclusion complex under different ball milling times. Table S2: chemical shift of H protons of DPG-HP-β-CD, HP-β-CD, and DPG. Table S3: the rebound rate and rebound height of DPG/NRLF, 0.2%, 0.3%, and 0.4%DPG-HP-β-CD/NRLF. Table S4: DPG/NRLF, 0.2%, 0.3%, and 0.4%DPG-HP-β-CD/NRLF crosslinking density results. [file 9814638.f1.docx]

Supplementary Information

Title

Design Strategy for Vulcanization Accelerator of Diphenylguanidine/Cyclodextrin Inclusion Complex for Natural Rubber Latex Foam with Enhancing Performance

**Authors**

Wang Zhang^1, 2†^, Liwei Lin^1†^*, Junqiang Guo^2^, Ming Wu^2^, Sumin Park^1^, Hang Yao^2^, Sun Ha Paek^3^, Guowang Diao^2^* and Yuanzhe Piao^1, 4^*

**Affiliations**

^1^ Department of Applied Bioengineering, Graduate School of Convergence Science and Technology, Seoul National University, Seoul, 08826, Republic of Korea

^2^ School of Chemistry and Chemical Engineering, Yangzhou University, Yangzhou, Jiangsu, 225002, China

^3^ Department of Neurosurgery, Movement Disorder Center, Seoul National University Hospital, Hypoxia/Ischemia Disease Institute, Cancer Research Institute, Seoul National University College of Medicine, Seoul, 03080, Republic of Korea

^4^ Advanced Institutes of Convergence Technology, 145 Gwanggyo-ro, Yeongtong-gu, Suwon-si, Gyeonggi-do, 16229, Republic of Korea

Correspondence should be addressed to Yuanzhe Piao; [parkat9@snu.ac.kr](mailto:parkat9@snu.ac.kr), Liwei Lin; [lin-official@snu.ac.kr](mailto:lin-official@snu.ac.kr), Guowang Diao; [gwdiao@yzu.edu.cn](mailto:gwdiao@yzu.edu.cn)

^†^ These authors contributed equally to this work.


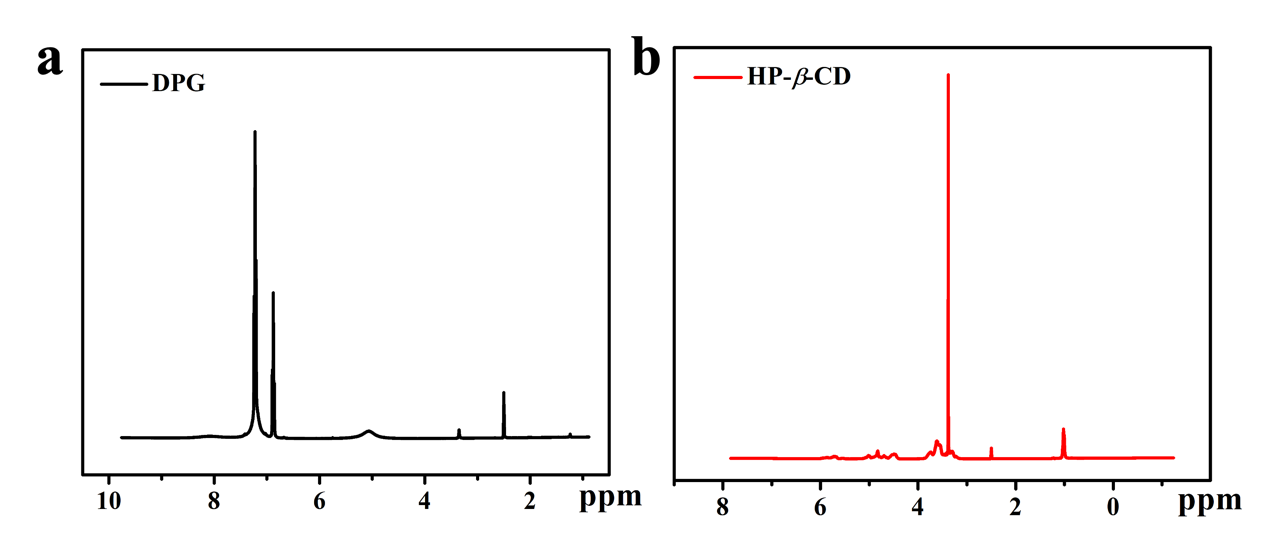


***Fig. S1.*** *^1^H NMR spectra of (a)DPG and (b) HP-β-CD.*


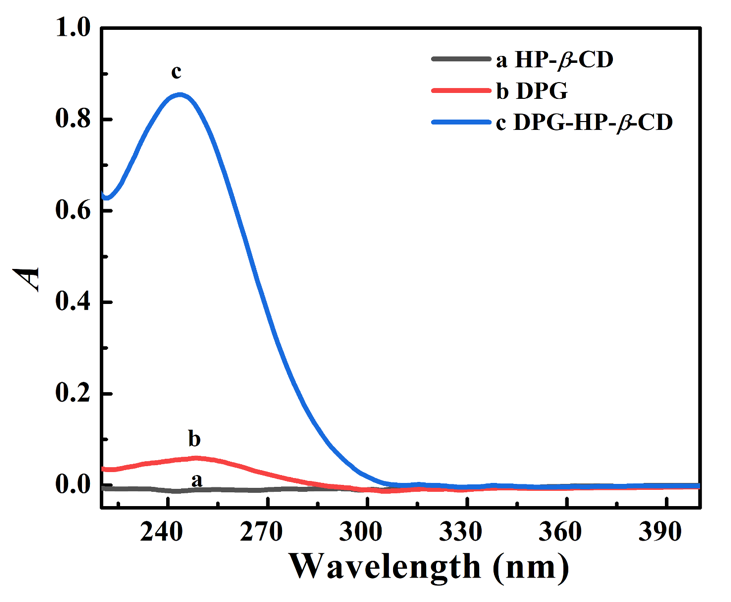


***Fig. S2.*** *UV spectra of HP-β-CD, DPG and DPG-HP-β-CD.*


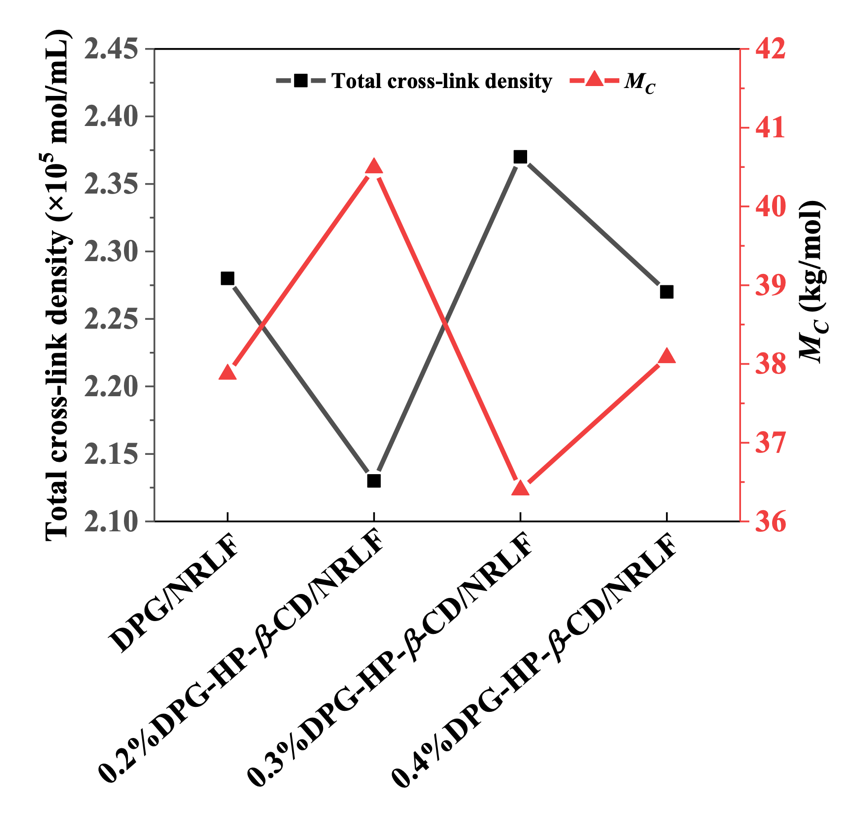


***Fig. S3.*** *Crosslinking density and relative molecular mass between crosslinking points.*


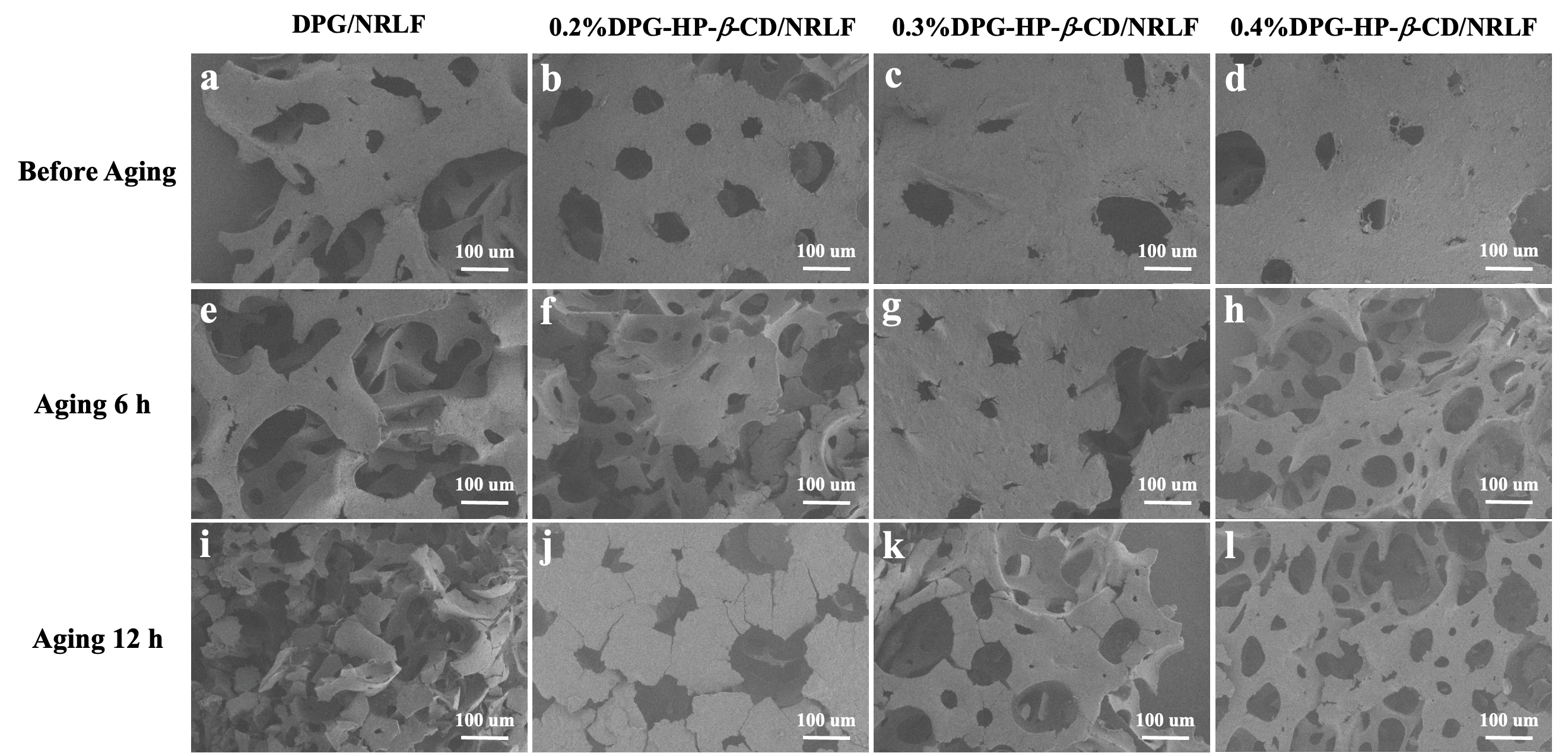


***Fig. S4.*** *SEM images of DPG/NRLF, 0.2%, 0.3% and 0.4%DPG-HP-β-CD/NRLF before aging (a-d), after aging 6 h (e-h) and aging 12 h at 100 ℃ (i-l).*


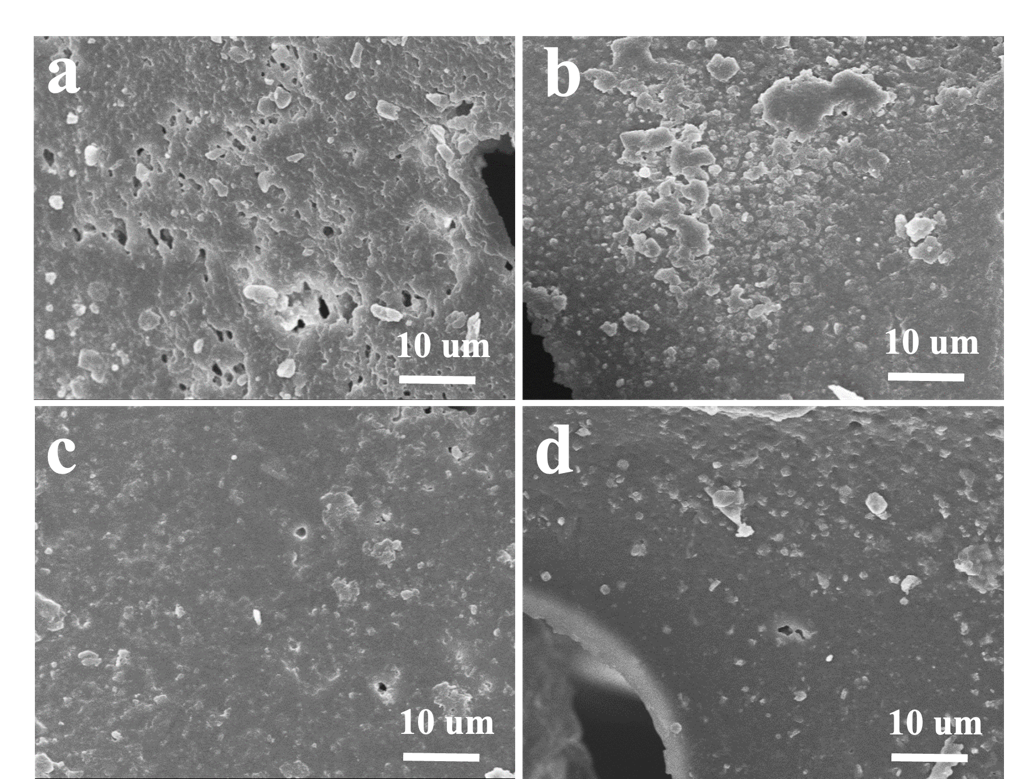


***Fig. S5.*** *SEM images of (a) DPG/NRLF, (b) 0.2%, (c) 0.3% and (d) 0.4%DPG-HP-β-CD/NRLF after aging 12 h at 100 ℃.*

***Table S1.*** *Contents of DPG in DPG-HP-β-CD inclusion complex under different ball milling time.*

| Sample | 1 | 2 | 3 | 4 | 5 |
| --- | --- | --- | --- | --- | --- |
| Time (min) | 20 | 40 | 60 | 80 | 100 |
| Encapsulation Efficiency (%) | 17 | 22 | 30 | 23 | 22 |

***Table S2.*** *Chemical shift of H protons of DPG-HP-β-CD, HP-β-CD and DPG.*

| Sample | H-1 | H-5,6 | H-a | H-b,c |
| --- | --- | --- | --- | --- |
| DPG |  |  | 6.88 | 7.22 |
| HP-β-CD | 5.71，5.86 | 3.74 |  |  |
| DPG-HP-β-CD | 5.74，5.90 | 3.75 | 6.92 | 7.24 |
| Δσ | 0.03，0.04 | 0.01 | 0.04 | 0.02 |

***Table S3.*** *The rebound rate and rebound height of DPG/NRLF, 0.2%, 0.3% and 0.4%DPG-HP-β-CD/NRLF.*

| Sample | Rebound Rate (%) | Rebound Height (mm) |
| --- | --- | --- |
| DPG/NRLF | 59.99 | 275.94 |
| 0.2%DPG-HP-β-CD/NRLF | 61.10 | 281.08 |
| 0.3%DPG-HP-β-CD/NRLF | 60.90 | 280.14 |
| 0.4%DPG-HP-β-CD/NRLF | 62.42 | 282.53 |

***Table S4.*** *DPG/NRLF, 0.2%, 0.3%, and 0.4%DPG-HP-β-CD/NRLF crosslinking density results.*

| Sample | T_2_ (ms) | qMrl | Cross-linking Specific Gravity (%) | Chain Specific Gravity (%) | Crosslinking Density (×10^5^ mol/mL) | M_C_  (kg/mol) |
| --- | --- | --- | --- | --- | --- | --- |
| DPG/NRLF | 6.26 | 3.76 | 72.93 | 27.07 | 2.28 | 37.87 |
| 0.2%DPG-HP-β-CD/NRLF | 6.48 | 3.29 | 73.79 | 26.21 | 2.13 | 40.49 |
| 0.3%DPG-HP-β-CD/NRLF | 7.8 | 4.07 | 60.99 | 39.01 | 2.37 | 36.40 |
| 0.4%DPG-HP-β-CD/NRLF | 7.45 | 3.72 | 65.02 | 34.98 | 2.27 | 38.08 |
